# Association and Diagnostic Performance of the Atherogenic Index of Plasma and Carotid Intima-Media Thickness in Non-alcoholic Fatty Liver Disease: A Cross-Sectional Study

**DOI:** 10.7759/cureus.105774

**Published:** 2026-03-24

**Authors:** Bharath V Karnati, Pradip Kumar Behera, Krishna P Tripathy, Rama Sowmya Karanam, Aparajita Priyadarshini, Swati Das, Subhashree Mishra

**Affiliations:** 1 Internal Medicine, Kalinga Institute of Medical Sciences, Bhubaneswar, IND; 2 Physiology, Kalinga Institute of Medical Sciences, Bhubaneswar, IND; 3 Radiodiagnosis, Kalinga Institute of Medical Sciences, Bhubaneswar, IND

**Keywords:** atherogenic index of plasma, cardiometabolic risk, carotid intima-media thickness (cimt), dyslipidemia, fatty liver index, masld, subclinical atherosclerosis

## Abstract

Background: Non-alcoholic fatty liver disease (NAFLD), recently termed metabolic dysfunction-associated steatotic liver disease (MASLD), is increasingly recognized as a multisystem metabolic disorder associated with significantly elevated risk of cardiovascular disease (CVD). Atherogenic Index of Plasma (AIP) and carotid intima-media thickness (CIMT) are considered as surrogate markers of atherosclerotic cardiovascular disease (ASCVD). As CVD is the common link between NAFLD and parameters, such as AIP and CIMT, the present study was conducted to determine any association and discriminatory ability of AIP and CIMT in relation to NAFLD severity.

Methods: This was a hospital-based cross-sectional study, conducted in the Department of General Medicine in collaboration with the Department of Radiodiagnosis at Kalinga Institute of Medical Sciences (KIMS), KIIT, Deemed to be University, Bhubaneswar, Odisha, India, from March 2023 to February 2025. A total of 151 participants (75 NAFLD cases and 76 controls) were enrolled during the study. NAFLD was graded by ultrasonography. AIP was calculated as log₁₀(triglyceride (TG)/high-density lipoprotein cholesterol (HDL)-C), Fatty Liver Index (FLI) was derived using the Bedogni formula, and CIMT was measured bilaterally using B-mode ultrasonography. In addition, correlation, multivariate regression, and receiver operating characteristic (ROC) curve analyses were performed.

Results: Among 151 participants (75 NAFLD cases and 76 controls), patients with NAFLD demonstrated significantly higher TG and lower HDL-C levels (p<0.001). The AIP was markedly elevated in patients with NAFLD compared to controls (0.82±0.24 vs. 0.42±0.17, p<0.001). Bilateral CIMT values were significantly greater in patients with NAFLD than in controls (right: 1.01±0.36 mm vs. 0.63±0.07 mm; left: 1.02±0.40 mm vs. 0.62±0.10 mm; p<0.001). Across ultrasound-defined NAFLD grades, TGs, AIP, and FLI increased progressively, whereas HDL-C declined significantly (p<0.001 for trend). FLI showed strong positive correlations with CIMT (right: ρ=0.607; left: ρ=0.663; p<0.001). Multivariate regression identified body mass index (BMI), TGs, HDL-C, AIP, and CIMT as independent determinants of NAFLD severity. The ROC analysis demonstrated excellent diagnostic performance for AIP (area under the curve (AUC) = 0.917) and combined AIP+CIMT (AUC = 0.951), indicating high discriminatory accuracy for identifying NAFLD.

Conclusions: NAFLD is strongly associated with increased atherogenic burden and subclinical carotid atherosclerosis. AIP and CIMT correlate with disease severity and demonstrate good diagnostic utility in identifying advanced NAFLD. These findings reinforce the systemic cardiometabolic nature of NAFLD and support the potential role of simple lipid indices and vascular imaging in early CVD risk stratification.

## Introduction

Non-alcoholic fatty liver disease (NAFLD), recently termed metabolic dysfunction-associated steatotic liver disease (MASLD), is now recognized as the most common cause of chronic liver disease worldwide, with an estimated global prevalence of 32.4%. In India, it affects 30% of the adult population [1,2]. NAFLD represents the hepatic component of metabolic syndrome, closely linked to obesity, dyslipidemia, and type 2 diabetes mellitus [3], which are established risk factors for cardiovascular disease (CVD).

MASLD, previously termed NAFLD, is increasingly recognized as a systemic cardiometabolic disorder with a complex bidirectional relationship with CVD. Shared pathophysiological mechanisms, including insulin resistance, chronic low-grade inflammation, oxidative stress, and endothelial dysfunction, contribute to both hepatic steatosis and accelerated atherosclerosis. Consequently, NAFLD/MASLD is strongly associated with increased risk of coronary artery disease, atrial fibrillation, heart failure, and cardiovascular mortality. Recent studies suggest that CVD represents the leading cause of death in individuals with steatotic liver disease, highlighting the systemic implications of hepatic metabolic dysfunction [4,5].

The pathophysiological link between steatotic liver disease and CVD is mediated through several interconnected metabolic pathways. Insulin resistance promotes hepatic triglyceride (TG) accumulation and increases circulating atherogenic lipoproteins. Concurrently, systemic inflammation, oxidative stress, and endothelial dysfunction impair vascular homeostasis and accelerate atherosclerotic plaque formation. These processes contribute to structural and functional vascular abnormalities that predispose patients with NAFLD/MASLD to coronary artery disease, heart failure, arrhythmias, and increased cardiovascular mortality [4,6].

The Atherogenic Index of Plasma (AIP), calculated as log₁₀ (TG/high-density lipoprotein cholesterol (HDL-C)), has emerged as a comprehensive marker of atherogenic dyslipidemia. Beyond indicating the presence of small dense low-density lipoprotein (LDL) particles, AIP is also associated with alterations in cholesterol esterification processes and the accumulation of TG-rich remnant lipoproteins, which contribute to endothelial dysfunction and atherosclerotic plaque formation [7]. AIP reflects the presence of small dense LDL particles, which are highly atherogenic due to their increased susceptibility to oxidation and enhanced arterial wall penetration [8]. Compared with isolated lipid parameters, AIP has demonstrated stronger associations with cardiovascular events, metabolic syndrome, and insulin resistance [9].

Carotid intima-media thickness (CIMT) is a validated, non-invasive marker of subclinical atherosclerosis [10] and an established surrogate indicator of future cardiovascular events. Measured using high-resolution B-mode ultrasonography, CIMT reflects early structural changes in the arterial wall that precede overt plaque formation. Increased CIMT has been shown to correlate with myocardial infarction and stroke risk in large population-based studies. Importantly, several investigations have reported higher CIMT values in individuals with NAFLD compared to metabolically healthy controls, suggesting accelerated vascular remodeling in this population [11]. However, data regarding the consistency of CIMT changes across different grades of NAFLD severity remain variable, particularly in Indian cohorts.

The Fatty Liver Index (FLI), derived from body mass index (BMI), waist circumference, TGs, and gamma-glutamyl transferase levels, provides a validated non-invasive estimate of hepatic steatosis severity [12]. FLI has been widely used in epidemiological studies and correlates with metabolic risk profiles. Exploring the relationship between FLI-based NAFLD severity and markers of subclinical atherosclerosis may offer additional insights into the metabolic-vascular axis.

Although previous studies have independently evaluated CIMT and AIP in metabolic disorders, limited data exist on examining their combined association with ultrasound-defined NAFLD severity in Indian populations. Furthermore, whether these markers demonstrate a graded relationship with NAFLD severity and FLI remains inadequately clarified. Identifying inexpensive, reproducible, and non-invasive markers that reflect both metabolic and vascular risk may facilitate early cardiovascular risk stratification in patients with NAFLD.

Therefore, the present study was undertaken to assess the association of CIMT and AIP with NAFLD and evaluate their relationship with disease severity in a hospital-based cross-sectional cohort.

## Materials and methods

Study design, setting, duration, and sample size

This hospital-based cross-sectional study was conducted in the Department of General Medicine in collaboration with the Department of Radiodiagnosis at Kalinga Institute of Medical Sciences (KIMS), KIIT Deemed to be University, Bhubaneswar, Odisha, India. The study period was from March 2023 to February 2025. Ethical clearance was obtained from the Institutional Ethics Committee.

The study by Wang et al., which considered an odds ratio of 5.37 for AIP at a 5% level of significance, 95% confidence interval, and 80% power, was used to calculate the minimum sample size of 148 [13]. A total of 151 participants (75 cases and 76 controls) were enrolled during the course of two years.

Subgroup analyses across NAFLD severity grades were performed exclusively among the 75 NAFLD cases. Controls were included only in case-control comparative analyses. Multivariate regression analysis evaluating determinants of fatty liver severity was restricted to patients with NAFLD.

Multivariate regression analysis was performed to determine independent associations, and receiver operating characteristic (ROC) curve analysis was performed to evaluate the discriminatory ability of AIP and CIMT for differentiating patients with NAFLD from controls. The combined cut-off was derived from logistic regression predicted probability using the Youden index.

The study was initiated before the 2023 international consensus redefining NAFLD as MASLD. Therefore, diagnostic criteria used in this study correspond to the previously established NAFLD definition, which substantially overlaps with MASLD criteria. However, the diagnostic variables required for MASLD classification were not fully captured, namely diabetes and hypertension. Therefore, the term "NAFLD" has been retained throughout the manuscript.

Study population

This hospital-based cross-sectional study included adult participants aged 18-60 years. Cases comprised patients diagnosed with NAFLD based on ultrasonographic findings, whereas controls included age-matched individuals with no evidence of liver pathology and a normal abdominal ultrasound examination. Only participants without significant comorbidities were enrolled, except for conditions specifically addressed in the exclusion criteria.

Individuals were excluded if they had a history of significant alcohol consumption (>30 g/day for men and >20 g/day for women), were pregnant, or had acute or chronic liver diseases, including cirrhosis or viral hepatitis. Patients with moderate-to-severe organ failure, rheumatological disorders, malignancies, metabolic liver diseases, such as Wilson’s disease or hemochromatosis, or drug-induced liver injury (including exposure to tamoxifen, glucocorticoids, methotrexate, or similar agents) were also excluded. Additional exclusions included patients receiving total parenteral nutrition, those who had undergone recent major surgery, and individuals with pre-existing significant CDV to minimize confounding related to vascular outcomes.

Anthropometric measurements

Height and weight were measured using calibrated hospital stadiometers and digital weighing scales, respectively, with participants wearing light clothing and no footwear. BMI was calculated as weight in kilograms divided by the square of height in meters (kg/m²).

Biochemical and radiological assessment

Blood samples were collected after overnight fasting and analyzed in the institutional central laboratory using standardized automated biochemical analyzers. Serum TGs and HDL cholesterol were measured using enzymatic colorimetric methods according to manufacturer protocols.

NAFLD severity was graded using B-mode ultrasonography by two experienced radiologists using a GE Voluson S10 B-mode ultrasound system (GE Healthcare; Chicago, IL, USA) to reduce inter-observer variability into Grade I (mild), Grade II (moderate), and Grade III (severe) [14]. The FLI was calculated using the Bedogni formula [12], and AIP was derived as log₁₀(TG/HDL-C) [7]. CIMT was measured 1 cm proximal to the carotid bifurcation bilaterally using a 7.5 MHz B-mode linear probe.

Statistical analysis

Data were analyzed using the IBM SPSS Statistics for Windows, version 26.0 (Released 2018; IBM Corp., Armonk, NY, USA). Continuous variables are expressed as mean±standard deviation (SD), and categorical variables as frequency and percentage. Normality was assessed using the Shapiro-Wilk test. Group differences were analyzed using the independent *t*-test or Mann-Whitney U test, and categorical data with chi-square or Fisher’s exact test. For comparisons of continuous variables across more than two independent groups (NAFLD Grades I, II, and III), one-way analysis of variance (ANOVA) was performed. Correlation between FLI, AIP, and CIMT was evaluated using Pearson’s or Spearman’s coefficient. Multivariate linear regression identified independent predictors, whereas ROC curve analysis assessed diagnostic performance using the area under the curve (AUC), sensitivity, and specificity. A p-value<0.05 was considered statistically significant.

## Results

Baseline clinical and biochemical characteristics

Baseline demographic, metabolic, and biochemical characteristics of NAFLD cases and healthy controls are displayed in Table 1*.* The mean ages of cases and controls were 48 and 49 years, respectively. Male NAFLD cases are predominantly higher than female cases. No significant differences were observed between cases and controls. BMIs of cases and controls were 28.06±3.94 kg/m^2^ and 25.9±4.42 kg/m^2^, respectively, and the difference was statistically significant. TG levels in cases were significantly higher than in the control group. HDL levels of NAFLD cases were significantly lower (30.57±8.06 mg/dL) than those of healthy controls (39.62±6.59 mg/dL). The AIP was significantly higher (0.82±0.24) under the high-risk category. Similarly, the FLI was revealed as a high-risk category (61.49±23.6). In comparison to healthy controls, the CIMT diameters were significantly higher in both the left and right sides (1.01±0.36 mm and 1.02±0.4 mm, respectively). 

**Table 1:** Comparison of Clinical and Biochemical Parameters Between NAFLD Cases (n=75) and Controls (n=76) BMI: body mass index; HDL-C: high-density lipoprotein cholesterol; CIMT: carotid intima-media thickness; SD: standard deviation

Parameter	Cases (n=75) (mean±SD)	Controls (n=76) (mean±SD)	p-value
Age (years)	49.72±8.06	48.17±9.97	0.2957
Gender (M/F) n (%)	45 (60%)/30 (40%)	48 (63.16%)/28 (36.84%)	0.83
Height (cm)	1.65±0.08	1.67±0.08	0.2762
BMI (kg/m²)	28.06±3.94	25.9±4.42	0.0018
Triglycerides (mg/dL)	205.68±70.52	105.49±33.19	<0.001
HDL-C (mg/dL)	30.57±8.06	39.62±6.59	<0.001
Atherogenic Index of Plasma (AIP)	0.82±0.24	0.42±0.17	<0.001
Fatty Liver Index (FLI)	61.49±23.6	30.14±18.3	<0.001
CIMT-right (mm)	1.01±0.36	0.63±0.07	<0.001
CIMT-left (mm)	1.02±0.4	0.62±0.1	<0.001

Comparison of metabolic and vascular parameters across NAFLD grades

All metabolic and vascular parameters across NAFLD grades in the cases are mentioned in Table 2*.* With increasing NAFLD severity from Grade I (n=45) to Grade 2 (n=24) and Grade III (n=6), a clear stepwise deterioration was observed in metabolic and vascular indices. Mean TG levels increased from 171.09±61.9 mg/dL in Grade I to 251.29±44.97 mg/dL in Grade II and 282.67+52.1 in Grade III (p<0.001). Conversely, HDL-C decreased progressively (35.02±6.21 → 25.54±4.92→ 17.33±1.21 mg/dL, p<0.001). AIP values increased sharply from 0.67±0.18 to 0.99±0.11 and 1.21+0.08 and FLI increased from 50.13±22.11 to 77±14.18 and 84.72±7.13 (p<0.001). Although metabolic parameters worsened significantly across NAFLD grades, CIMT measurements did not show statistically significant variation between ultrasound grades, with CIMT-right and CIMT-left decreasing from 1.02±0.42 mm and 1.01±0.44 mm in Grade I to 1.02±0.12 mm and 0.97±0.16 mm in Grade III, respectively. Although CIMT was significantly higher in patients with NAFLD compared with controls, CIMT did not demonstrate a statistically significant stepwise increase across ultrasound-defined grades of steatosis in our cohort. This finding may reflect the fact that CIMT represents cumulative vascular injury related to metabolic dysfunction rather than short-term variation in hepatic fat severity. The analysis was restricted to patients with NAFLD only.

**Table 2:** Biochemical and Vascular Parameters Across NAFLD Grades (Among NAFLD Cases Only, n=75) FLI: Fatty Liver Index; HDL-C: high-density lipoprotein cholesterol; AIP: Atherogenic Index of Plasma; CIMT: carotid intima-media thickness; SD: standard deviation; NAFLD: non-alcoholic fatty liver disease; ANOVA: analysis of variance

Parameter	Grade I (n=45) mean±SD	Grade II (n=24) mean±SD	Grade III (n=6) mean±SD	p (ANOVA)
Triglycerides (mg/dL)	171.09±61.9	251.29±44.97	282.67±52.1	<0.001
HDL-C (mg/dL)	35.02±6.21	25.54±4.92	17.33±1.21	<0.001
AIP	0.67±0.18	0.99±0.11	1.21±0.08	<0.001
FLI	50.13±22.11	77±14.18	84.72±7.13	<0.001
CIMT-right (mm)	1.02±0.42	0.99±0.26	1.02±0.12	0.9376
CIMT-left (mm)	1.01±0.44	1.06±0.39	0.97±0.16	0.8416

Multivariate regression analysis for predictors of NAFLD severity

Multivariate regression analysis for predictors of NAFLD severity is shown in Table 3*.* 

**Table 3:** Multivariate Linear Regression Analysis for Independent Associations of NAFLD Severity (Among NAFLD Cases Only, n=75) HDL-C: high-density lipoprotein cholesterol; AIP: Atherogenic Index of Plasma; CIMT: carotid intima-media thickness; BMI: body mass index; NAFLD: non-alcoholic fatty liver disease

Independent variable	β	Standard error	t-value	p-value
BMI (kg/m²)	0.274	0.061	4.49	<0.001
Triglycerides (mg/dL)	0.232	0.057	4.07	<0.001
HDL-C (mg/dL)	-0.198	0.063	-3.15	0.002
AIP	0.345	0.071	4.86	<0.001
CIMT-right (mm)	0.312	0.069	4.52	<0.001
CIMT-left (mm)	0.29	0.069	4.02	<0.001

Multivariate modeling identified several independent associations of the FLI. BMI (β=0.274, standard error (SE)=0.061, t=4.49, p<0.001), TGs (β=0.232, SE=0.057, t=4.07, p<0.001), and HDL-C (β=-0.198, SE=0.063, t=-3.15, p=0.002) were significant metabolic predictors. Among composite indices, AIP (β=0.345, SE=0.071, t=4.86, p<0.001) and CIMT-right (β=0.312, SE=0.069, t=4.52, p<0.001) remained robust independent determinants of NAFLD severity. Collectively, these variables accounted for a substantial proportion of variance in FLI, confirming the intertwined roles of adiposity, dyslipidemia, and vascular wall thickening in the pathophysiology of NAFLD. The analysis was restricted to patients with NAFLD only [15].

Correlation between FLI and CIMT

Correlation analysis showed that FLI was significantly and positively correlated with both carotid wall measurements. For the right CIMT, Spearman’s ρ was 0.607 (p<0.001), whereas it was 0.663 (p<0.001) for the left CIMT. These strong positive coefficients confirm that higher fatty-liver indices correspond to greater carotid-intima thickening, underscoring a close link between hepatic lipid accumulation and early atherosclerotic vascular remodeling. The bilateral correlation pattern indicates that systemic metabolic inflammation in NAFLD contributes symmetrically to vascular wall hypertrophy.

Diagnostic performance of AIP and CIMT (ROC curve analysis)

**Figure 1:**
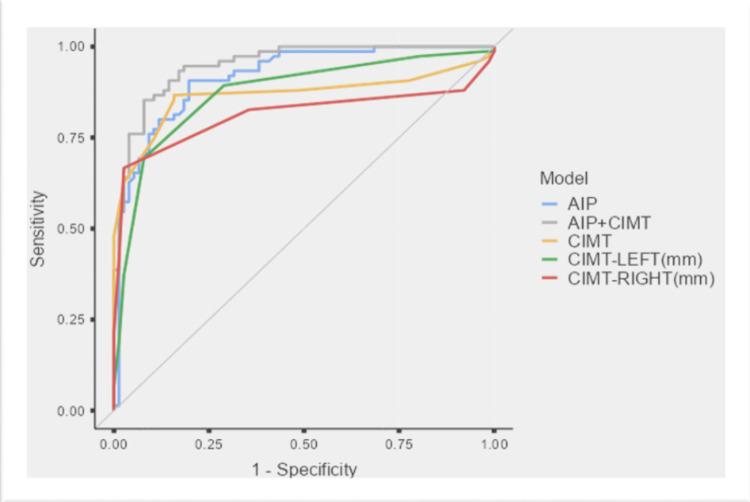
Receiver Operating Characteristic (ROC) Curve Analysis of AIP, CIMT, and Combined Model for Identifying NAFLD AIP: Atherogenic index of plasma; CIMT: carotid intima-media thickness; NAFLD: non-alcoholic fatty liver disease

The ROC curve analysis results for AIP and CIMT in predicting NAFLD severity are mentioned in Table 4 and Figure 1*.*

**Table 4:** ROC Curve Analysis for AIP and CIMT in Identifying NAFLD AIP: Atherogenic index of plasma; CIMT: carotid intima-media thickness; ROC: receiver operating characteristic; NAFLD: non-alcoholic fatty liver disease; AUC: area under the curve; CI: confidence interval

Variable	AUC (95% CI)	Cut-off	Sensitivity (%)	Specificity (%)	p-value
AIP	0.917 (0.873-0.961)	0.49	90.67	80.26	<0.001
CIMT-right (mm)	0.813 (0.736-0.889)	0.75	66.67	97.37	<0.001
CIMT-left (mm)	0.873 (0.816-0.930)	0.75	69.33	92.11	<0.001
Combined (AIP+CIMT)	0.951 (0.920-0.981)	1.25	85.33	92.11	<0.001

ROC evaluation revealed excellent discriminatory accuracy of both AIP and CIMT parameters. The AIP demonstrated an AUC of 0.917 (95% CI=0.873-0.961), with a cut-off ≥0.49 yielding 90.67% sensitivity and 80.26% specificity (p<0.001). For CIMT-right and CIMT-left, AUCs were 0.813 (0.736-0.889) and 0.873 (0.816-0.930), respectively, at thresholds of 0.75 mm and 0.75 mm, providing 66.67% and 69.33% sensitivity and 97.37% and 92.11% specificity. When AIP and CIMT were combined, the correlation increased further (AUC=0.951 (0.920-0.991), p<0.001). These findings highlight the complementary diagnostic value of lipid-based and vascular parameters in differentiating patients with NAFLD from controls [16].

## Discussion

NAFLD is increasingly recognized as a systemic cardiometabolic disorder rather than an isolated hepatic condition. In the present study, patients with NAFLD exhibited a significantly higher atherogenic burden and evidence of subclinical vascular remodeling compared with metabolically healthy controls. Notably, these associations were observed in the absence of overt CVD, supporting the concept that NAFLD functions as an early cardiovascular risk amplifier.

NAFLD and atherogenic dyslipidemia: beyond conventional lipid measures

The lipid profile observed in patients with NAFLD demonstrated significantly elevated TG and reduced HDL-C levels compared with controls. Rather than relying solely on absolute lipid parameters, the AIP provided an integrated reflection of this pro-atherogenic shift. Elevated AIP values in patients with NAFLD indicate predominance of small dense LDL particles, which are more susceptible to oxidative modification and arterial wall penetration.

These findings are consistent with those of Younossi et al. and Dobiásová et al., who highlighted AIP as a surrogate marker of LDL particle size and a strong predictor of CVD in metabolic disorders [1,7]. The progressive increase in AIP across ultrasound-defined grades of steatosis further demonstrated a dose-response relationship between hepatic fat accumulation and worsening atherogenic risk. Similar observations by Bedogni et al. and Kim et al. support the mechanistic link between dyslipidemia, hepatic insulin resistance, oxidative stress, and vascular injury [12,17].

Age may further modulate the relationship between the AIP and hepatic steatosis. Advancing age is associated with progressive insulin resistance, increased visceral adiposity, and alterations in lipid metabolism that favor elevated TG levels and reduced HDL-C levels. These metabolic changes contribute to a higher AIP and promote hepatic lipid accumulation through increased delivery of free fatty acids to the liver and impaired lipid oxidation. Consequently, older individuals may exhibit a stronger association between atherogenic dyslipidemia and severity of hepatic steatosis, reflecting the cumulative metabolic burden over time [1,11].

The components of the API, namely elevated TGs and reduced HDL-C, reflect key metabolic disturbances involved in hepatic steatosis. Increased circulating TGs are associated with enhanced hepatic very-low-density lipoprotein (VLDL) production and impaired lipid clearance, promoting TG deposition within hepatocytes. Conversely, reduced HDL levels impair reverse cholesterol transport and anti-inflammatory vascular functions. In metabolic states characterized by dyslipidemia, activation of nuclear receptors, such as liver X receptors (LXR), can stimulate transcription of lipogenic genes, leading to increased de novo hepatic lipogenesis and accumulation of lipid droplets within hepatocytes, thereby contributing to the development and progression of fatty liver disease [7,11]. Collectively, these findings reinforce the clinical utility of AIP as an inexpensive, scalable marker for early cardiometabolic risk stratification in NAFLD.

CIMT: a marker of systemic vascular injury

CIMT, a validated surrogate marker of early atherosclerosis, was significantly higher in patients with NAFLD compared with controls, supporting previous work by Tilg and Moschen, who demonstrated a close association between hepatic steatosis and subclinical atherosclerosis [6].

Although CIMT did not show a strict stepwise increase across all ultrasound grades of steatosis, Spearman's correlation analysis revealed a robust positive association between hepatic fat indices and CIMT values. This aligns with the findings from the study by Wang et al., who reported that increasing fatty liver indices correspond with measurable increments in carotid wall thickness [13].

The absence of strict grade-wise escalation may suggest that structural vascular changes plateau once systemic metabolic inflammation and endothelial dysfunction are established. CIMT likely reflects cumulative cardiometabolic exposure rather than short-term variations in hepatic fat severity.

FLI as a metabolic-vascular bridge

The FLI, derived from routine anthropometric and biochemical parameters, demonstrated strong correlations with both AIP and CIMT. This provides mechanistic coherence: FLI reflects the metabolic environment driving hepatic steatosis, which concurrently promotes endothelial dysfunction and vascular remodeling.

The bilateral association between FLI and CIMT supports the concept that hepatic lipid accumulation and carotid wall thickening are parallel manifestations of a shared systemic process rather than isolated pathologies. These findings are in agreement with those of previous meta-analyses by Suresh et al. and Lorenz et al., who reported increased subclinical atherosclerosis in patients with NAFLD [4,10].

Independent predictors of NAFLD severity

Multivariate regression analysis identified BMI, TGs, HDL-C, AIP, and CIMT as independent predictors of NAFLD severity. These variables collectively represent the metabolic-vascular axis underlying NAFLD progression. The convergence of adiposity, dyslipidemia, and vascular remodeling reinforces contemporary models that describe NAFLD as the hepatic expression of metabolic syndrome.

Diagnostic performance and clinical implications

The ROC analysis demonstrated excellent discriminatory performance of AIP and CIMT in identifying NAFLD. When used in combination, predictive accuracy improved further, suggesting that laboratory-based lipid indices and ultrasonographic vascular markers complement one another.

The high sensitivity and specificity observed in this study highlight the potential utility of integrating AIP and CIMT into routine clinical evaluation of patients with NAFLD. This strategy may facilitate early identification of individuals at increased cardiovascular risk, particularly in resource-limited settings where advanced imaging or liver biopsy is not feasible.

Pathophysiological integration

Mechanistically, insulin resistance promotes hepatic overproduction of TG-rich lipoproteins and increases the circulation of small dense LDL particles. These particles penetrate the arterial intima, triggering oxidative stress and inflammatory cascades that accelerate vascular remodeling. Simultaneously, reduced HDL-mediated reverse cholesterol transport impairs vascular repair mechanisms. The concurrent elevation of FLI, AIP, and CIMT observed in this study reflects a shared inflammatory and metabolic substrate rather than independent disease pathways.

Clinical and preventive perspective

Given the strong association between NAFLD and subclinical atherosclerosis, early cardiometabolic screening is warranted even in patients without established CDV. Incorporating low-cost, non-invasive indices, such as AIP and FLI, alongside CIMT assessment may enhance cardiovascular risk stratification and guide early preventive interventions. Lifestyle modification remains the cornerstone of management in patients with MASLD and associated cardiometabolic risk. Dietary approaches, such as the Dietary Approaches to Stop Hypertension (DASH) diet, have demonstrated beneficial effects on hepatic steatosis, metabolic syndrome components, and cardiovascular outcomes. The DASH diet emphasizes consumption of fruits, vegetables, whole grains, and unsaturated fats while reducing sodium and saturated fat intake. Emerging evidence suggests that adherence to DASH dietary patterns may improve insulin sensitivity, reduce systemic inflammation, and lower the risk of hypertension, diabetes, and cardiovascular mortality in patients with metabolic liver disease [4]. Timely lifestyle modification, metabolic optimization, and appropriate pharmacotherapy may mitigate long-term cardiovascular morbidity in this high-risk population.

Limitations

This study has several limitations that warrant consideration. First, the cross-sectional design precludes inference of causality between NAFLD severity, atherogenic dyslipidemia, and vascular remodeling. Longitudinal studies are required to determine whether worsening hepatic fat accumulation directly accelerates vascular structural changes over time.

Secondly, CIMT measurement, although validated as a surrogate marker of subclinical atherosclerosis, more advanced vascular imaging modalities, such as coronary artery calcium scoring or carotid plaque assessment, may provide complementary prognostic information.

Third, selection bias may have occurred because participants were recruited from a single tertiary care center, which may limit the generalizability of the findings to the broader population and affect the external validity of the study. Larger multicenter studies would strengthen external validity. Furthermore, the sample size, although adequate to demonstrate statistically significant associations, may limit subgroup analyses and the detection of smaller effect sizes, particularly across different grades of steatosis.

Finally, although ultrasound is widely used as a first-line screening modality due to its accessibility and cost-effectiveness, it has recognized limitations, including reduced sensitivity in detecting mild steatosis (<20-30% hepatic fat), operator dependence, and limited ability to quantify hepatic fat content or fibrosis. Recent advances have introduced several non-invasive modalities such as transient elastography, controlled attenuation parameter (CAP), magnetic resonance imaging-proton density fat fraction (MRI-PDFF), and serum biomarker-based scores, which provide more accurate assessment of hepatic steatosis and fibrosis. Future studies incorporating these advanced diagnostic techniques may further improve the precision of NAFLD/MASLD assessment [18,19].

## Conclusions

NAFLD is associated with increased atherogenic burden and subclinical carotid atherosclerosis. AIP is strongly associated with NAFLD severity and may be utilized as an inexpensive marker to assess it. Similar to AIP, CIMT can be used as a non-invasive marker to evaluate fat burden in cases of NAFLD.
